# Supplementary material for: CRISPR-Cas12a for Highly Efficient and Marker-Free Targeted Integration in Human Pluripotent Stem Cells
Source: Int J Mol Sci. 2024 Jan 12;25(2):985. doi: 10.3390/ijms25020985 (PMC10816062; doi:10.3390/ijms25020985)
Supplement: Supplementary file 1 [file ijms-25-00985-s001.zip › ijms-2792217-supplementary.pdf]

Supplementary Figures

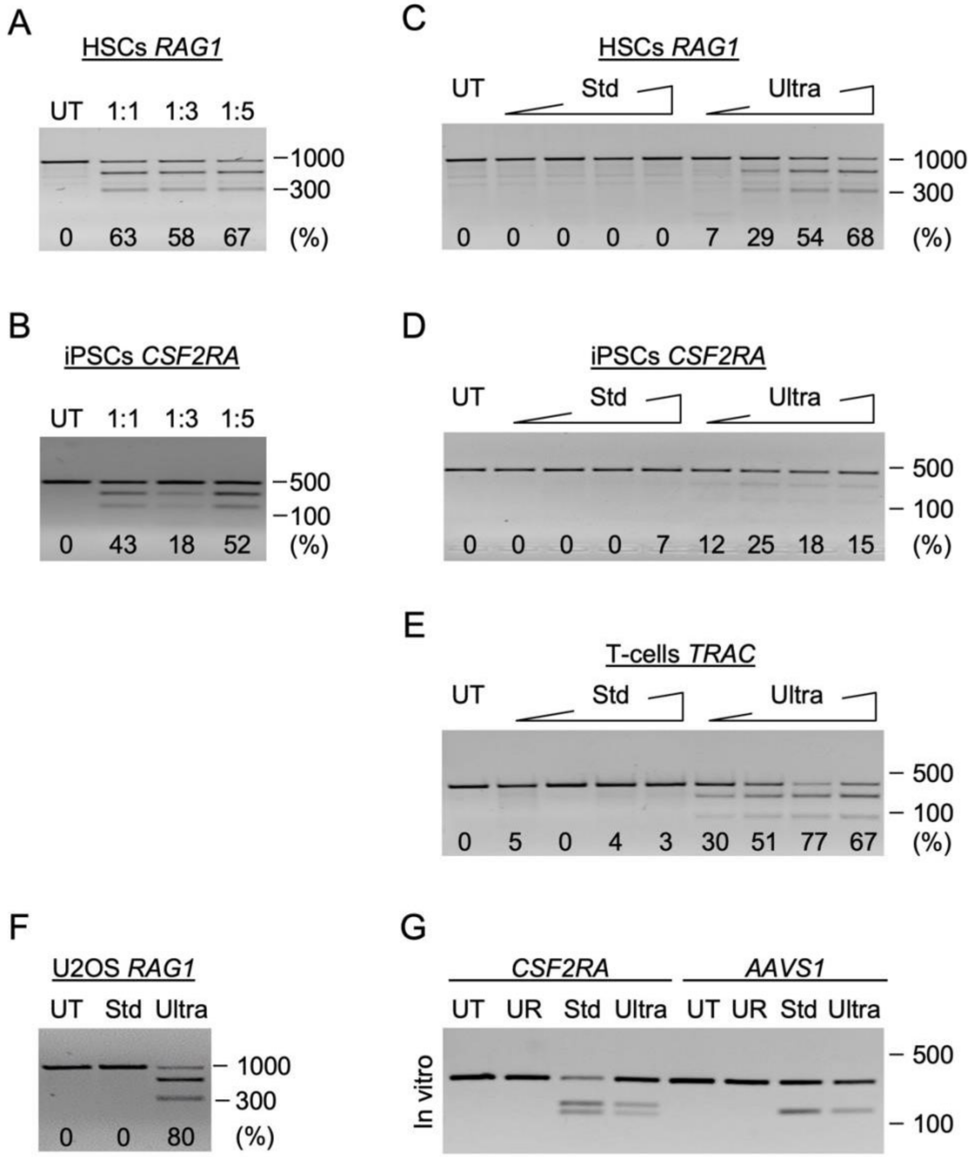

Figure S1. Assessment of CRISPR-Cas12a nuclease activity. (A-B) Titration of Cas12a Ultra to crRNA ratio. Primary cells (A, HSPC; B, iPSCs) were nucleofected with the indicated Cas12a Ultra to crRNA ratios. The mutagenic CRISPR-Cas12a activity was determined by T7E1 assay. The fraction of cleaved product is indicated. **(C-E)** Titration of CRISPR-Cas12a amount. Primary cells (C, HSC; D, iPSCs, E, T cells) were nucleofected with increasing amounts (0.75 μg, 1.5 μg, 3 μg, 6 μg) of standard CRISPR-Cas12a or CRISPR-Cas12a Ultra. The mutagenic activity of the nucleases was determined by T7E1 assay. The fraction of cleaved product is indicated. **(F)** CRISPR-Cas12a activity in U2OS cells. Cells were nucleofected with either standard CRISPR-Cas12a or CRISPR-Cas12a Ultra. The mutagenic activity was determined by T7E1 assay. The fraction of cleaved product is indicated. **(G)** *In vitro* cleavage. Indicated targets were PCR amplified, subjected to *in vitro* cleavage with either standard Cas12a or Cas12a Ultra nuclease, and then evaluated by agarose gel electrophoresis. Unrelated guided RNA (UR) and untreated target fragments (UT) were used as controls. UT, untreated; Std, standard Cas12a nuclease; Ultra, Cas12a Ultra nuclease.

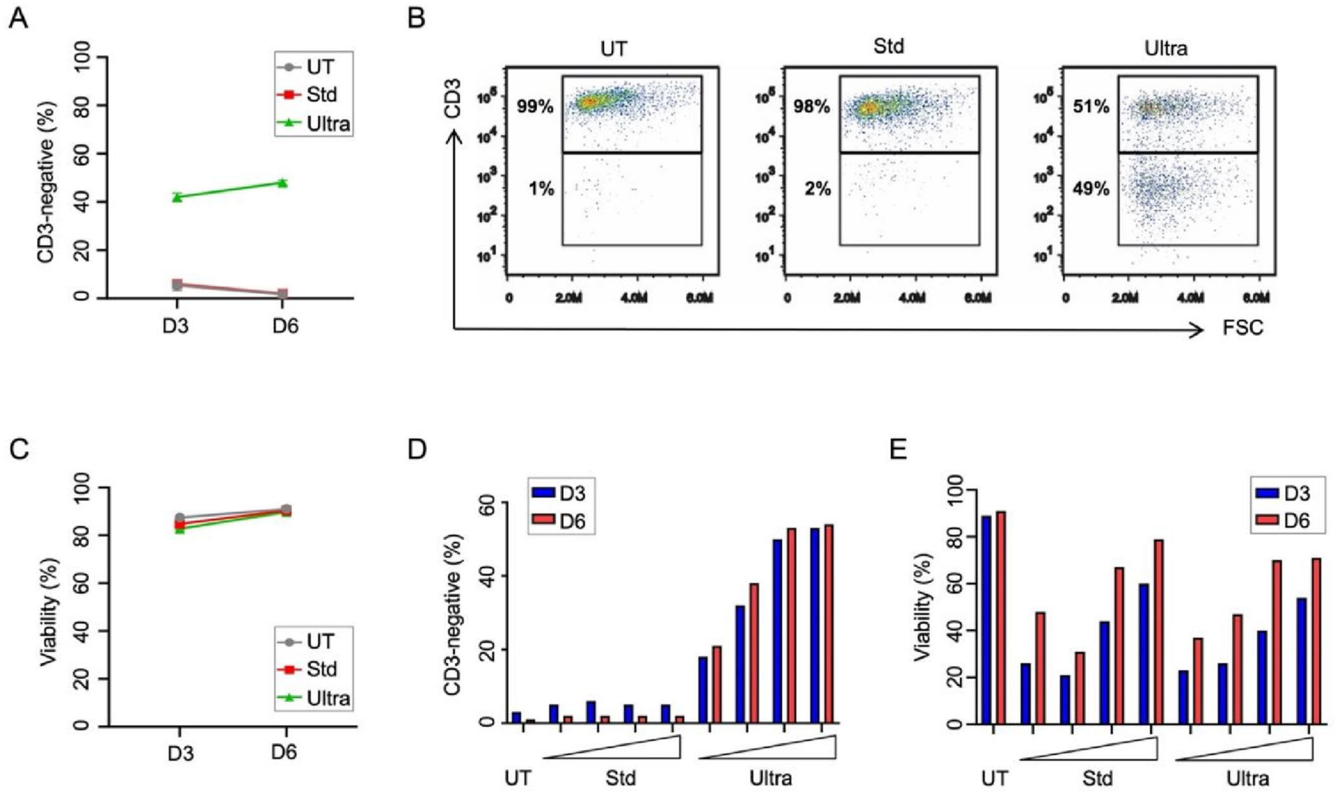

Figure S2 CRISPR-Cas12a Ultra mediated knockout in primary T cells. The *TRAC* locus in T cells was edited with either standard CRISPR-Cas12a or CRISPR-Cas12a Ultra. **(A-C)** Phenotypic analysis. At days 3 and 6 after nucleofection, T cells were evaluated by flow cytometry and on an automated cell counter. The average fraction of CD3-negative cells (A), the original plots for a sample measured at day 6 post-nucleofection (B), and cell viability (C) are shown. N=4; 2 independent donors. **(D-E)** Impact of CRISPR-Cas12a dose. T cells were nucleofected with increasing amounts (0.75 µg, 1.5 µg, 3 µg, 6 µg) of standard CRISPR-Cas12a or CRISPR-Cas12a Ultra. The fraction of CD3-negative cells was determined by flow cytometry (D), while cell viability was evaluated on an automated cell counter (E). UT, untreated cells; Std, standard Cas12a nuclease; Ultra, Cas12a Ultra nuclease.

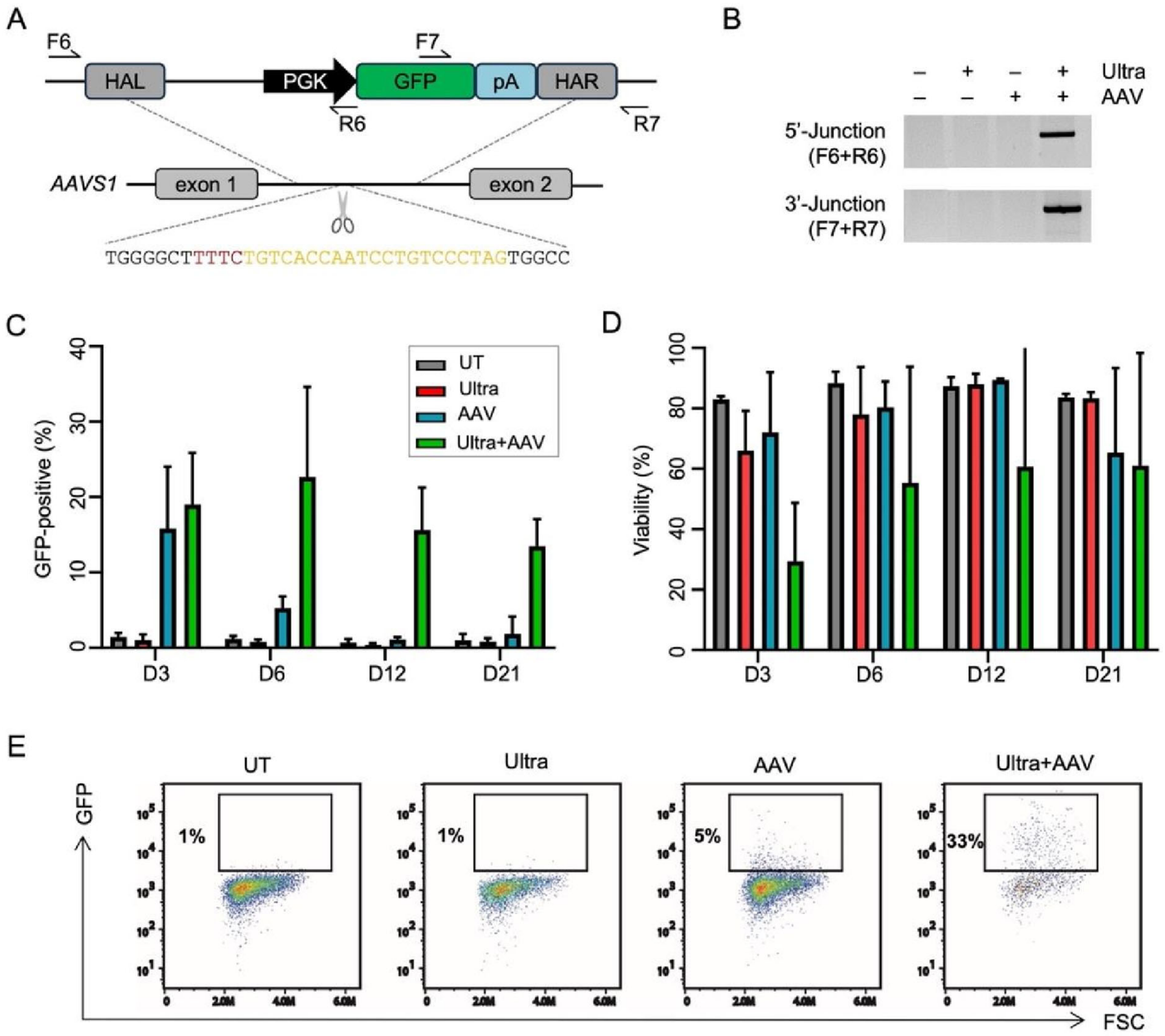

Figure S3 Knock-in of *GFP* into *AAVS1* safe harbor site in T cells. (A) Schematic of gene targeting strategy. Shown are the *AAVS1* target site and the AAV6-based homology-directed repair (HDR) template. Relevant elements of the HDR template comprise a PGK promoter driven GFP transgene followed by a Herpes simplex virus type-1 poly adenylation signal (pA) which is flanked by homology arms left (HAL) and right (HAR). **(B)** Genotyping. T cells nucleofected with *AAVS1*-targeted CRISPR-Cas12a Ultra nuclease were transduced with the AAV6 HDR template. Targeted integration was detected by 5'- and 3'-junction PCRs using the indicated primers. **(C-E)** Phenotyping. The percentage of GFP-positive T cells (C) and cell viability (D) was quantified by flow cytometry at indicated time points. A representative flow cytometric evaluation is shown (E). N=3 independent experiments. UT, untreated T cells; AAV; AAV6 only; Ultra, CRISPR-Cas12a Ultra only; Ultra+AAV, T cells nucleofected with CRISPR-Cas12a Ultra and transduced with AAVs HDR template; F, forward primer; R, reverse primer; PGK, phosphoglycerate kinase promoter.

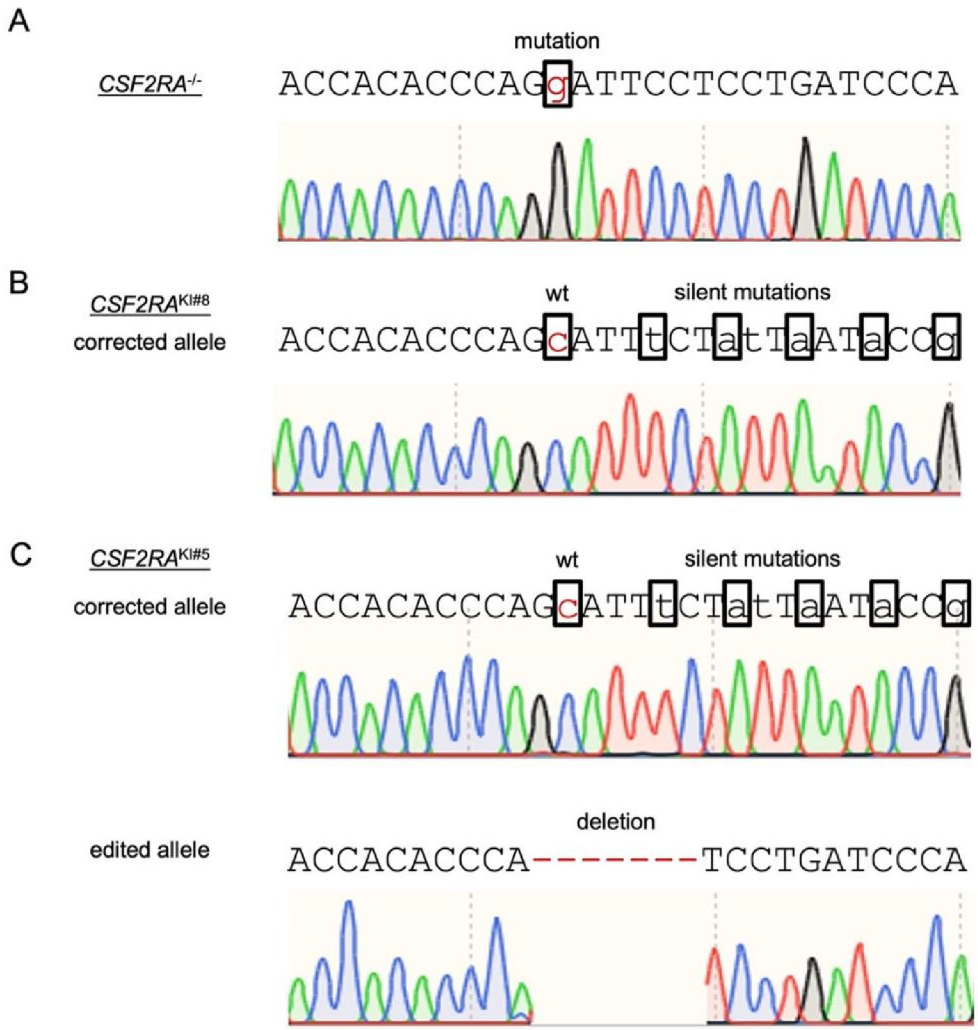

Figure S4 Sequence of *CSF2RA* knock-in clones. Shown are the chromatograms aligned to the reference sequences. Red letters indicate the position of the A17G mutation which was changed to a C in the integrated super-exon. Black boxes with lower-case letters indicate the silent mutations introduced in the *CSF2RA* HDR template**. (A)** Original clone. **(B)** Clone *CSF2RA*$^{KI\#5}$. **(C)** Clone *CSF2RA*$^{KI\#8}$.
